# FRET Based Biosensor: Principle Applications Recent Advances and Challenges

**DOI:** 10.3390/diagnostics13081375

**Published:** 2023-04-08

**Authors:** Awadhesh Kumar Verma, Ashab Noumani, Amit K. Yadav, Pratima R. Solanki

**Affiliations:** Lab D NanoBiolab, Special Centre for Nanoscience, Jawaharlal Nehru University, New Delhi 110067, India

**Keywords:** FRET, fluorophore, fluorescent QDs, foster radius, biosensor

## Abstract

Förster resonance energy transfer (FRET)-based biosensors are being fabricated for specific detection of biomolecules or changes in the microenvironment. FRET is a non-radiative transfer of energy from an excited donor fluorophore molecule to a nearby acceptor fluorophore molecule. In a FRET-based biosensor, the donor and acceptor molecules are typically fluorescent proteins or fluorescent nanomaterials such as quantum dots (QDs) or small molecules that are engineered to be in close proximity to each other. When the biomolecule of interest is present, it can cause a change in the distance between the donor and acceptor, leading to a change in the efficiency of FRET and a corresponding change in the fluorescence intensity of the acceptor. This change in fluorescence can be used to detect and quantify the biomolecule of interest. FRET-based biosensors have a wide range of applications, including in the fields of biochemistry, cell biology, and drug discovery. This review article provides a substantial approach on the FRET-based biosensor, principle, applications such as point-of-need diagnosis, wearable, single molecular FRET (smFRET), hard water, ions, pH, tissue-based sensors, immunosensors, and aptasensor. Recent advances such as artificial intelligence (AI) and Internet of Things (IoT) are used for this type of sensor and challenges.

## 1. Introduction

### 1.1. What Is the Importance of FRET in Biosensor?

FRET is frequently used in biosensor devising because it allows for the specific and sensitive detection of biomolecules in a highly specific manner with high sensitivity without the need for modification of the biomolecule or direct labeling. The fluorescence of the acceptor molecule is only activated when both the donor and acceptor fluorophore molecule are in close juxtaposition, so any changes in the environment that affect the distance between the two molecules will also affect the fluorescence. This allows for the detection of small changes in the environment, such as the presence of a specific biomolecule, without the need to directly label or modify that biomolecule [1,2]. Additionally, FRET is a non-radiative process, which means that it does not produce any ionizing radiation. This makes FRET-based biosensors safer to use and handle than other types of biosensors that rely on radioactive or ionizing radiation [3]. Furthermore, FRET biosensors are highly specific, meaning that they can detect a specific biomolecule or change in the environment without being affected by other molecules or changes that might be present. This specificity is achieved by designing the biosensor to have a high binding affinity for the target biomolecule and by engineering the donor and acceptor molecules to be close to each other [4]. FRET biosensors are also highly sensitive and versatile, as they can detect a wide range of biomolecules and changes in the environment and can be used in a variety of applications, such as detecting protein–protein interactions, monitoring changes in pH, measuring the activity of enzymes, etc. [5,6,7,8,9,10,11,12]. FRET is a photo-physio-chemical, quantum mechanical, distance-dependent, non-radiative transfer of energy from the photon excited donor fluorophore to a suitable electron-acceptor fluorophore in ground state when both the fluorophores are close to each other (1–10 nm). It is also called FRET in honor of the discovery of a phenomenon by German scientist Theodor Förster [13]. It is a widely accepted accessory tool to quantify molecular dynamics for biomolecular interactions such as protein–DNA interactions, protein–protein interactions, and protein conformational changes in biochemistry and biophysics. In order to monitor the conjugate formation between two different molecules, FRET is labeled with a donor and acceptor fluorophore molecule, respectively. When these fluorophore-labeled molecules are dissociated after the mixing, the emission of donor gets detected upon the donor excitation. Additionally, emission of the acceptor is predominantly observed due to intermolecular FRET between the donor and acceptor due to their interaction when they are in proximity (1–10 nm) with each other. To monitor the conformational changes in protein, the target protein molecule is labeled with a donor as well as an acceptor molecule at two loci. FRET changes are observed upon twisting or bending of protein molecules. This occurs due to relative orientation or distance change between donor and acceptor fluorophores. If change in protein conformation or molecular interaction is associated with binding of ligand, this technique is useful for ligand detection via fluorescent indicators [14,15,16]. Researchers can use FRET to measure the distance between the two parts and infer whether the protein is in an active or inactive state.

Apart from protein interaction and protein detection, FRET is also extensively used for quantitative detection in polymerase chain reaction (PCR) [3]. In PCR, a pair of short synthetic DNA primers are used to amplify a specific region of DNA. The primers bind to the target DNA sequence and provide a starting point for the amplification process [4]. FRET can be used as a detection method for PCR. In this technique, one of the PCR primers is labeled with a donor fluorophore, and the other primer is labeled with an acceptor fluorophore. When the two primers bind to the target DNA sequence and are in proximity, the donor fluorophore transfers its energy to the acceptor fluorophore, resulting in a fluorescent signal [5]. FRET can be used in real-time PCR to monitor the amplification of DNA as it occurs [6]. As the PCR reaction progresses, the amount of amplified DNA increases, which leads to an increase in FRET signal. This allows for the quantification of the amount of DNA present in the reaction [7]. Various fluorophores can be used as donors and acceptors in FRET-based PCR [8]. It can also be called real time quantitative PCR, including fluorescein, rhodamine, and cyanine dyes. The specific choice of fluorophores will depend on factors such as the instrument used for detection and the specific requirements of the experiment. The article “A novel coronavirus outbreak of global health concern” published in Eurosurveillance in January 2020 describes the use of FRET in PCR for the detection of the novel coronavirus (SARS-CoV-2) responsible for the COVID-19 pandemic. The authors used a FRET-based assay targeting two regions of the SARS-CoV-2 genome, the E gene and the RdRp gene, to detect the virus in respiratory specimens from patients with suspected COVID-19. The assay consisted of two sets of primers labeled with different fluorescent dyes, and a probe that binds to a specific sequence between the primers. During PCR amplification, the probe binds to the target sequence and is cleaved by the Taq polymerase, which releases the two fluorescent dyes. The resulting increase in FRET signal can be detected in real time using a fluorescence-based PCR instrument. The authors reported that the FRET-based assay showed high sensitivity and specificity for the detection of SARS-CoV-2, with a limit of detection of 10 copies of viral RNA per reaction. They also noted that the FRET-based assay could be easily adapted for high-throughput testing in clinical laboratories. Overall, the use of FRET in PCR has become a valuable tool for the detection and monitoring of SARS-CoV-2, as well as for a wide range of other infectious agents. The high sensitivity and specificity of FRET-based assays make them ideal for the rapid and accurate detection of pathogens in clinical and research settings [9]. In 2022, Zhang et al. developed a nucleic acid biosensor for the rapid detection of SARS-CoV-2 viral sequence. In this paper, the authors used FRET concept for the detection of viral sequence. In the given experiment, they selected ssDNA as a donor motif and 2D nanomaterials as an acceptor motif [10].

For nano–bio system also, FRET is a reliable, accurate, and highly sensitive tool to monitor nano–bio interaction at ultra-low concentration, i.e., nanomolar and picomolar level [17]. FRET is also helpful to monitor the complex cellular events, dynamics, and interactions of biological systems in vitro as well as in vivo. The results gained in life science research are analyzed by numerous established approaches. FRET is very much crucial in terms of understanding the interactions of nano-systems with biomolecules for efficient use of nanotechnology. On the contrary, understanding the enzyme kinetics at lower concentration of enzymes is highly crucial to understand the mechanism that could be possible by the use of fluorescent QDs probing. Despite several limitations, FRET is still a highly accurate and sensitive tool to monitor such type of interactions [18,19,20,21,22].

### 1.2. Importance over Other Conventional Technique

It is very difficult to monitor the interaction between the biomolecule at very short distance (less than 100 nm), which makes FRET technique dominant over the other conventional technique. FRET also has an advantage over the other technique in terms of the monitoring of reactions at ultra-low concentration [17]. This is because FRET measures energy transfer between molecules, rather than the amount of light emitted. FRET is a non-invasive technique that does not require any chemical modification of the molecules being studied. This means that the molecules can retain their natural properties, and the results obtained from FRET studies are more representative of the actual biological processes [11]. FRET can be used to monitor dynamic changes in molecular interactions in real time. This is particularly useful for studying biological processes such as protein–protein interactions, DNA replication, and signal transduction. FRET has a high spatial resolution and can measure distances between molecules within a few nanometers [12]. This makes it possible to study molecular interactions within specific cellular compartments or even within single cells. FRET can be used to study a wide range of biological processes and interactions, including protein–protein interactions, protein–DNA interactions, and protein–lipid interactions [13,14]. Several techniques have their own advantages and limitations, but FRET has an extra corner on these techniques such as SPR, ELISA, electrochemical biosensor, mass spectrophotometry, colorimetric assays fluorescence microscopy, impedance biosensors, and quartz crystal microbalance.

### 1.3. Importance of FRET in Biosensor

Biosensing of drug molecules, biomolecules, and chemical compounds at ultra-low concentration is very much crucial in biochemical research. FRET-based sensors and biosensors based on fluorescent material enables us to deal with such types of challenges, especially quantum dots probe-based biosensors. Nowadays, FRET has become the most powerful spectroscopic technique to detect such molecules including heavy metal ions and transition elements with very high sensitivity at picomolar and nanomolar concentration [23]. FRET is an important technique in biosensors because it allows for the specific and sensitive trace of biomolecules without the need for direct labeling or modification of the biomolecule. This allows for the detection of small changes in the environment, such as the presence of a specific biomolecule, without the need to directly label or modify that biomolecule. FRET biosensors are also highly specific, meaning that they can detect a specific biomolecule or change in the environment without being affected by other molecules or changes that might be present. This specificity is achieved by designing the biosensor to have a high binding affinity for the target biomolecule, and by engineering the donor and acceptor molecules to be in adjoined proximity to each other. Sensitivity of FRET biosensors is also very high, often in the picomolar range, making them able to detect very low amounts of biomolecules, which is especially important for early detection of diseases or for detecting low levels of environmental pollutants. FRET biosensors are also highly versatile, as they can detect a wide range of biomolecules and changes in the environment, and can be used in a variety of applications, such as detecting protein–protein interactions, monitoring changes in pH, or measuring the activity of enzymes. This versatility makes FRET-based biosensors a valuable tool for research, diagnosis, and monitoring in various fields, including biochemistry, cell biology, and drug discovery. In recent days, the FRET-based biosensor has touched almost every aspect of research from physiology to pathology, from diagnosis up to treatment, and from health to environment [24].

### 1.4. Detection of Conformational Changes Using FRET

**Figure 1:**
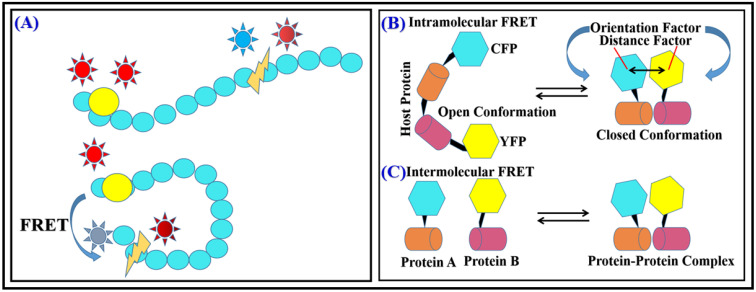
(**A**) FRET during protein conformational change. (**B**) Intramolecular (**C**) Intermolecular FRET.

FRET is a hihgly powerful tool for detecting conformational changes in biomolecules because it is sensitive to distance-dependent changes between the FRET donor–acceptor pair. When a conformational change occurs in a biomolecule, the distance amid the FRET donor–acceptor pair can change, which can affect the efficiency of energy transfer between them. By measuring the FRET efficiency, researchers can infer the conformation of the biomolecule. FRET is widely used in structural biology, molecular biology, and biochemistry to study the structural dynamics of biomolecules and their interactions with other molecules in real time and in a non-invasive way. For example, by attaching a FRET donor and acceptor molecule to different parts of a protein of interest, researchers can use FRET to measure the distance between the two parts and infer whether or not the protein is in an active or inactive state. Similarly, by using FRET, researchers can detect conformational changes in nucleic acids, lipids, and other biomolecules (Figure 1A) [15,16].

### 1.5. Determination of Inter and Intramolecular Interactions

One of the most common applications of FRET for detecting intermolecular interactions is to use it to measure the distance between two or more biomolecules. Intermolecular interactions involve the binding of one molecule to another, and they are crucial for many biological processes such as signal transduction, gene regulation, and protein–protein interactions. By attaching a FRET donor and acceptor molecule to different parts of the biomolecules of interest, researchers can use FRET to measure the distance between them. If the biomolecules are not interacting, the distance between the FRET donor and acceptor will be large, leading to low FRET efficiency. However, if the biomolecules bind to each other, the distance decreases between the donor–acceptor pair, leading to a higher FRET efficiency. FRET can also be used to detect intramolecular interactions within a single biomolecule. Intramolecular interactions involve the binding of different parts of the same molecule, and they are crucial for many biological processes such as enzyme catalysis, protein folding, and regulation of protein activity. For example, by attaching a FRET donor and acceptor molecule to different domains of a protein, researchers can use FRET to measure the distance between the domains. If the domains are not interacting, the distance between the FRET donor–acceptor pair will be large, leading to low FRET efficiency. However, if the domains bind to each other, the distance between both will decrease, resulting in higher FRET efficiency. Another way to detect intramolecular interactions by FRET is to use it to monitor changes in the conformation of a biomolecule caused by the interaction of different domains within the same molecule. For example, by attaching a FRET donor and acceptor molecule to different parts of a protein, researchers can use FRET to monitor changes in the conformation of the protein caused by the interaction of different domains. This constitutes FRET as a primal tool for screening inter- as well as intramolecular interactions along with conformational changes that help to understand the mechanisms of reactions. FRET is used to study numerous bimolecular interactions such as protein–DNA interaction and interaction between proteins, enzyme kinetic reaction, changes in reaction kinetics, bonding conformation, and configurationally changes. Reaction rate can also be detected by FRET as transfer efficiency is very much distance dependent, with charge-transfer property and dipolar orientations of biomolecules (Figure 1B,C) [17,18,19].

### 1.6. Enzyme Kinetic Studies through FRET

FRET can also be used to study enzyme kinetics, i.e., rate at which enzymes catalyze reactions. Enzyme kinetics provides important information on how enzymes work and how they can be regulated, which is crucial for understanding many biological processes. This is possible beacause of spectral overlapping J(λ) of emissin of donor fluorophore and absorption of acceptor fluorophore. This varies as interaction-dependent transfer of energy at all levels of the reaction kinetics. One way to use FRET for enzyme kinetic studies is to attach a FRET donor and acceptor molecule to different parts of an enzyme, such as the active site or a regulatory domain. By measuring the FRET efficiency, researchers can infer the conformation of the enzyme and how it changes during the course of the reaction. This can provide insights into the mechanism of enzyme catalysis and how the enzyme interacts with substrates and inhibitors. FRET-based methods are very useful in enzyme kinetic studies because they allow researchers to monitor the dynamics of enzyme–substrate and enzyme–inhibitor interactions in real time and in a non-invasive way. This can provide important insights into enzyme mechanisms, regulations, and drug discovery. Additionally, FRET can monitor the rate of product formation proteases and nucleases by using suitable substrates as donor [20].

## 2. FRET Description Details

### 2.1. Principle of FRET

**Figure 2:**
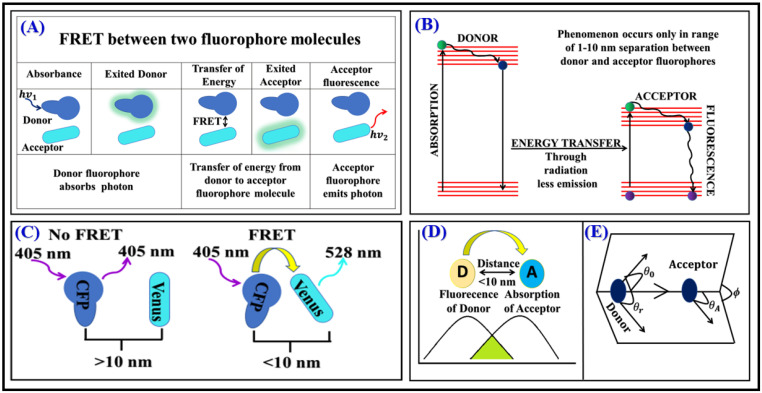
(**A**) Energy transfer between two fluorophore molecules. (**B**) Jablonski diagram for donor–acceptor pair in FRET. (**C**) Orientation of donor–acceptor pair, parallel to each other for maximum transfer of energy represented by spectral overlapping of emission spectra of donor (**D**) and absorption spectra of an acceptor (**A**). (**D**) Overlapping integral during FRET and (**E**) dipole–dipole orientation for maximum transfer of energy.

FRET stands for fluorescence resonance energy transfer. It is an extremely used photo-physiochemical phenomenon that works in a distance-dependent manner. In this phenomenon, quantum mechanical and non-radiative transfer of energy takes place from a photon-excited donor fluorophore to the suitable electron acceptor fluorophore in ground state. It occurs through dipole and dipole interaction, when both fluorophore molecules are in close juxtaposition [21] The basic principle of FRET is based on the transfer of energy from a donor to an acceptor molecule through dipole–dipole interaction and non-radiative energy transfer via time–space. The energy transfer occurs only when the donor and acceptor molecules are close enough, typically within 1–10 nm, and when their electronic energy levels are well matched [22]. The donor fluorophore molecule must first be excited by absorbing light photon of suitable frequency (E = hυ) at a specific wavelength. Once excited, it can transfer some of its energy to the acceptor molecule if the two are close enough. This transfer of energy leads to a decrease in fluorescence emission from the donor molecule and an increase in fluorescence emission from the acceptor molecule. The efficiency of the energy transfer is dependent on the distance between the donor and acceptor molecules, their spectral overlap, and the orientation of the molecules relative to each other. Figure 2B shows a Jablonski diagram of a donor–acceptor pair showing FRET. It is a visual representation of the energy transfer and fluorescence processes in FRET. It illustrates the energy levels and transitions between them for a donor and acceptor molecule. The Jablonski diagram has four energy levels: ground state (S_0_), first excited singlet state (S_1_), first excited triplet state (T_1_), and a non-fluorescent ground state (S_0_) after emission. When the donor molecule absorbs light, it is excited to the first excited singlet state (S_1_). From here, it can either return to the ground state (S_0_) through spontaneous emission, leading to fluorescence, or through FRET, where it can transfer its energy to the acceptor molecule. In FRET, the energy is transferred from the excited donor molecule (S_1_) to the ground state acceptor molecule (S_0_), which is then excited to the first excited singlet state (S_1_). After the transfer, the donor molecule returns to the ground state (S_0_) without emitting fluorescence. The acceptor molecule then returns to the ground state (S_0_) through fluorescence. The Jablonski diagram below helps to explain the FRET efficiency, which mainly depends on spectral overlapping between the donor and acceptor fluorophore, distance, and the orientation of the fluorophore molecules (Figure 2) [22,23,24,25,26,27,28,29,30,31,32,33].

### 2.2. Donor and Acceptor Fluorophores

Donor is the fluorophore molecule that emits radiation when excited with the photon having suitable frequency and wavelength. It gives up its energy through non-radiative transfer to an acceptor molecule. The donor molecule can be a fluorescent protein, fluorescent nanomaterials such as quantum dots, a fluorescent dye, or any other type of molecule that can transfer energy through FRET. Acceptor is a fluorphore molecule that receives energy transferred by a donor molecule. The acceptor molecule can be a fluorescent protein, fluorescent quantum dots, a fluorescent dye, or any other type of molecule capable of accepting energy through FRET [34].

### 2.3. Necessary Conditions for FRET

Fluorophore molecules primarily follow the conditions such as dipole orientation, distance, spectra, spectral overlapping, and lifetime of the donor for the occurrence of FRET to occur. FRET can only occur if the distance between donor fluorophore and acceptor is within the 1–10 nm range. Donor emission spectrum must overlap with acceptor absorption spectrum, i.e., ground-excited state energy difference of donor–acceptor fluorophore molecules should be comparable. The dipolar orientation of the donor–acceptor pair must be parallel to each other for maximum transfer of energy. Lifetime of the donor should be greater than the acceptor [16,31].

### 2.4. Spectral Overlap

**Figure 3:**
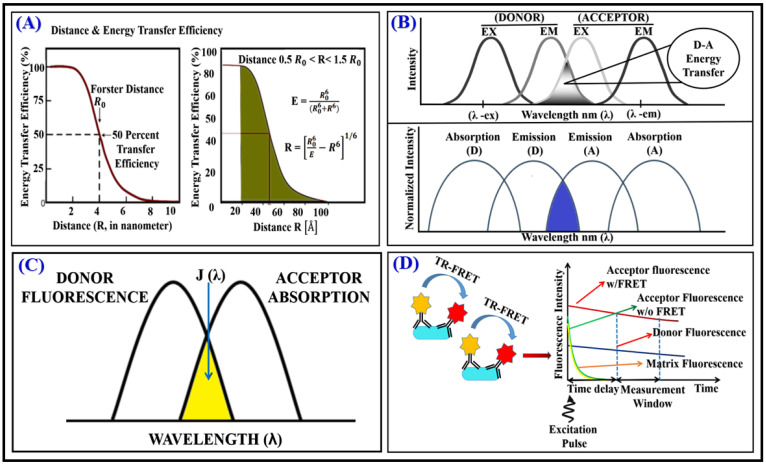
(**A**,**B**) Overlap integral for donor fluorescence and acceptor absorption. (**C**) Transfer efficiency. (**D**) TR-FRET.

Spectral overlap refers to the extent of overlapping between the emission and absorption spectrum of a donor and acceptor molecule, respectively, in FRET. It is the crucial factor in determining the capacity of energy transfer between the donor and acceptor. The greater the spectral overlapping, the more energy will be transferred by the donor to the acceptor. Spectral overlap is dependent on the wavelengths of light emitted by the donor and absorbed by the acceptor, as well as the distance between both the molecules. A high degree of spectral overlap is required for efficient FRET to occur, and this is typically achieved by selecting donor and acceptor molecules with complementary emission and absorption spectra. Figure 3A shows overlap donor (D) fluorophore spectrum with acceptor (A) fluorophore absorption. Overlap integral *J*(*λ*) for energy transfer is denoted by (1).

(1) $$J\left(\lambda \right)=\frac{\int_{0}^{\infty }F_{D}\left(\lambda \right)\cdot \varepsilon \cdot {\left(\lambda \right)}^{4}d\lambda }{\int_{0}^{\infty }F_{D}\left(\lambda \right)\cdot d\lambda }$$

where *J*(*λ*) (M^−1^ cm^−1^ nm^4^) is the spectral overlap integral between emission spectrum of donor and absorption spectrum of acceptor, *ε* symbolizes extinction coefficient of acceptor (M^−1^ cm^−1^), and *F_D_* is the fluorescence intensity of donor within wavelength range *λ* to *λ* + *dλ* [35].

In this review article, we are mentioning here some of the commercial donor–acceptor pairs which are used in FRET. They are detailed as follows. mCerulean3/mVenus: This donor–acceptor pair was introduced by Nagai and colleagues in 2002 [36] and is now available as a commercial FRET sensor from Clontech (now Takara Bio). Clover/mRuby2: This donor–acceptor pair was introduced by Lam and colleagues in 2012 [37] and is now available as a commercial FRET sensor from Evrogen. Citrine/mRuby2: This donor–acceptor pair was introduced by Miyawaki and colleagues in 2003 [38] and is now available as a commercial FRET sensor from Clontech. ECFP/YPet: This donor–acceptor pair was introduced by Miyawaki and colleagues in 2000 [38] and is now available as a commercial FRET sensor from Clontech. GFP/mCherry: This donor–acceptor pair was introduced by Shaner and colleagues in 2004 [38] and is now available as a commercial FRET sensor from Clontech.

The threshold of spectral overlap required for efficient FRET transfer between a donor and acceptor fluorophore depends on various factors, such as the distance and orientation between the fluorophores, the quantum yield and lifetime of the donor and acceptor, and the spectral properties of the donor and acceptor, among others. In general, a higher spectral overlap between the donor emission and acceptor absorption spectra is expected to result in more efficient FRET transfer. There is no specific threshold of spectral overlap that applies universally to all FRET pairs. However, a study by Wallrabe and Periasamy (2005) analyzed the spectral overlap requirements for various FRET pairs based on Monte Carlo simulations and experimental measurements. They found that a spectral overlap integral (J) of at least 40 nm^4^ was required for efficient FRET transfer in most cases, with higher J values corresponding to higher FRET efficiencies. However, they noted that some FRET pairs with lower J values could still exhibit efficient FRET transfer due to other factors such as favorable fluorophore orientations [39].

### 2.5. FRET Measurement

Measurement of FRET may be performed either by measuring the decrease in fluorescence intensity of a donor molecule or by measuring the increase in PL intensity of an acceptor molecule. Generally, intensity should be normalized in order to avoid error as well as measurement dependency [40].

### 2.6. Förster Radius

The Förster radius is a measure of the maximum distance between a donor and an acceptor molecule in FRET for efficient energy transfer. It is the distance at which the energy transfer efficiency between both molecules is half of its maximum value. It is dependent on the spectral overlap between the emission spectrum of the donor and the absorption spectrum of the acceptor, as well as the transition dipole moments of the donor and acceptor molecules. The Förster radius is a prerequisite parameter in determining the proficiency of FRET and is used to optimize the design of FRET-based biosensors. Equation (2) is the theoretical equation to calculate the Förster radius (R_0_) [35].

(2) $$R_{0}=\;{\left[\frac{Q_{D}J\left(\lambda \right)\left\{9000\;\left(\ln10\right)\right\}\kappa^{2}}{{128\pi }^{5}{µ}^{4}N_{A}}\right]}^{1/6}$$

where R_0_ depicts Förster radius, Q_D_ is the quantum yield, and κ^2^ is the orientation/coupling component for fluorophores and it is 2/3 in case of random orientation. µ is the R.I that is refractive index of medium and N_A_ is Avogadro’s number.

### 2.7. FRET Efficiency

FRET efficiency is a measure of the efficiency of energy transfer between both molecules in FRET. It is a dimensionless quantity that indicates the fraction of energy that is transferred from the donor to the acceptor. It is dependent on several factors, including the spectral overlap between the emission spectrum and absorption spectrum of both the donor and acceptor, respectively, the distance between both molecules, and transition dipole moments of the donor as well the acceptor. High FRET efficiency is desirable in FRET-based biosensors, as it allows for accurate measurement of biological processes. The FRET efficiency can be optimized by adjusting the donor and acceptor molecule properties, such as the emission and absorption spectra, or by changing the geometry of donor–acceptor molecules to reduce the distance between them. It is generally varying as reciprocal of 6th power of distance between both pairs shown in Equation (3) [41].

E_FRET_ = R_0_^6^/(R_0_^6^ + R^6^) (3)

Figure 3 shows the transfer of maximum energy at R_0_ that is the inverse ratio to the 6th power of distance between both the molecules. R_0_ is a distance, where 50% energy is transferred. R represents donor–acceptor pair distance as shown in Figure 3. Energy transfer may be estimated by the use of intensity of fluorescence of the donor (I_D_) in the absence of an acceptor and intensity of fluorescence (I_DA_), when donor–acceptor remain in medium as described by Equation (4).

E_FRET_ = 1 − (I_DA_/I_D_) (4)

### 2.8. Classical Methods for FRET Measurement

#### 2.8.1. Organic Dyes as Fluorescent Probes in FRET

Organic dyes such as fluoresceine, cyanines, rhodamines, etc., are mainly used as fluorescent probes in FRET due to their versatility, high brightness, and well-defined spectral properties. In FRET, organic dye is typically used as the donor or acceptor molecule. The choice of both depends on the specific requirements of the experiment, such as the spectral overlap between both molecules, the transition dipole moments of the molecules, and the distance between the donor and acceptor. Organic dyes can also be chemically modified to improve their performance as FRET probes, such as reducing their photobleaching rates and improving their water solubility [42].

#### 2.8.2. Fluorescent Proteins as Probes in FRET

Fluorescent proteins are a valuable tool for FRET experiments due to their excellent spectral properties, stability, and suitability. Many biologically related events and a wide category of signals contribute towards a broad range of emission and absorption spectra [43] as compared to organic dyes. Naturally occurring proteins are found in many organisms, including bacteria, plants, and animals. Fluorescent proteins have several advantages over organic dyes as FRET probes. For example, they are highly stable and exhibit little to no photobleaching, which makes them ideal for long-term imaging experiments. Fluorescent proteins are also well suited for live-cell imaging, as they are not toxic to cells and do not interfere with cellular processes. There are several protein pairs that act as a standard donor–acceptor pair such as blue-green fluorescence proteins, cyan-yellow, etc. Mutant varieties are also used to modify colors, which can serve as a basic tool that helps to investigate some intricate processes involved in living cells. GFP (green fluorescent protein) has been popularly used for almost a decade. In recent days, efforts are being made continuously for novel fluorescent proteins (FP) fabrication that may have exclusive features such as excellent excitation as well as emission wavelengths along with increase in brightness. FRET occurs between two different types of fluorescent proteins also known as FP-FRET, which is mainly utilized for sensors in the field of biomolecules. This type of sensor has been used for assays of intracellular cyclic adenosine monophosphate (cAMP) activity, protease activity, and Ca ions. Intermolecular FRET detects protein and protein interactions such as oligomerization of receptors transcription factors (TF). However, proteins show the broad spectra of absorption–emission, and therefore exhibit the prominent amount of cross-talk which might puddle the abstraction [19,44,45].

#### 2.8.3. Drawback of Organic Dye and Fluorescent Protein Fluorophore in FRET

With non-symmetric emission spectrum and low amount of absorption co-efficient, organic fluorophores exhibit the narrow absorption spectra. Acridone organic dyes have short lived fluorescence (except a few dyes) in the near IR and visible region and life time ~1 ns.

Applications of FP have some drawbacks because of the mixfixation process with tissue or the molecules, emission spectrum, and broad absorption leading to the low emission intensity and cross-talk, due to several reasons. In order to counter the limitations, a new kind of fluorescent material of congenial size is needed as probing material for several FRET approaches, leading to better efficiency, higher yield, and sensitivity. In recent years, Shashi et al. explained how upconversion nanoparticles (UCNPs) are used as a donor due to their unique optical properties. UCNPs can also convert low energy to high emission energy. UCNPs nowadays are mainly used in FRET bioanalysis. In this experiment, they used UCNP-to-dye FRET DNA-hybridization assays in H_2_O and D_2_O using ~24 nm large NaYF4:Yb^3+^,Er^3+^ UCNPs coated with thin layers of silica (SiO_2_) or poly(acrylic acid) (PAA). The results showed that UCNPs-to-dye FRET translated into microRNA FRET assay with LOD of 100 fmol [46]. Nan et al. in 2022 discussed the overall properties and uses of several types of nanoparticles in light of the fluorescent nano-biosensor technique for the detection of cancer biomolecular markers. In this review article, they were trying to understand and compile the different nanoparticles and their advantages and disadvantages for FRET-based biosensor [47]. In the nanotechnology era, fluorescent QDs are promising materials that can replace oraginc dyes and fluoroscent protein [42,48].

#### 2.8.4. Fluorescent QDs Based FRET

There are several advantages of fluorescent quantum dots such as sharp emission, enhanced brightness, photo and chemical stability, resistance to photo-bleaching, long PL lifetime, broad range of absorption spectra, narrow emission peak with symmetry, and about 10 to 100 times greater molar extinction coefficient [31]. QDs show very high efficiency and quantum yield. Band gap of semiconductor QDs can be tailored according to the need that can give several colors of the emission from ultraviolet to infrared range. It exhibits longer lifetime as compared to organic dye. As we know, QDs provide a wide range of excitation wavelength that differs from acceptor fluorophores. FRET based on fluorescent QDs is used to make nano-biosensors that are used for various purposes such as sensing of enzyme activity, identification of polypeptides and low molecular weight compounds of nucleic acid, microorganisms, and toxins produced along with environmental pollutants. This can also be used as pH sensors, immunoassay, and single molecular detection of protein. Imaging using fluorescent QDs conquers the FP limitations. Higher PL intensities entitle the QDs for better imaging at the molecular level that make it suitable for in vitro and in vivo imaging [49,50,51,52,53,54,55].

#### 2.8.5. Fluorescent QDs as an Efficient Probe in FRET

**Figure 4:**
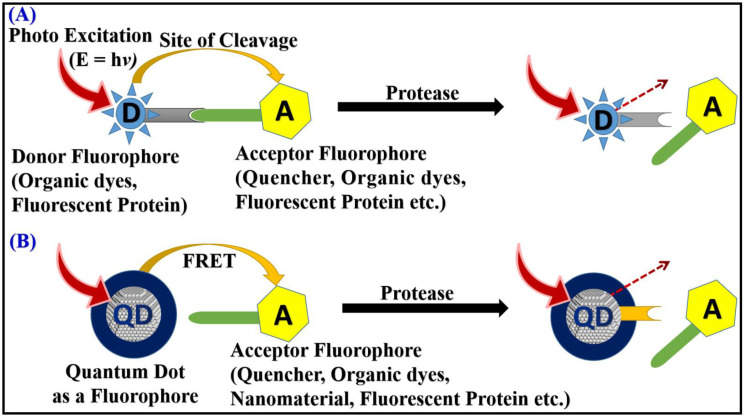
Protease activity detection using FRET: (**A**) old and conventional approach; (**B**) FRET based on fluorescent QDs.

Fluorescent QDs are majorly being used in applications based on FRET. Polymer capped semiconductor, core shall, polymer capped, bio molecular conjugated, and metal QDs are synthesized for several FRET-based biological applications. Core-shell CdSe/ZnS QDs show very high quantum that can be used as fluorescence-based bio-probing for ultra-sensitive single molecular detection [56,57]. CsdSe-QDs based on FRET [38] also explain protease merriment. Mercaptopropyl acid modified CdTe QDs can be used to analyze the immuno-complex via FRET (Figure 4).

#### 2.8.6. Merits and Demerits of Fluorescent QDs Probe Used in FRET

QDs are non-toxic, mostly biocompatible, and stable within a particular range of concentration. They are also able to sense the alter deportment of cells. This entitles, in particular, cases such as to understand the characters of cancer or tumor cells. QDs can function as a probe drug to cure the problem and drug carriers. When QDs work as fluorophores, they exhibit strong emission and longer life, which can easily overcome the several problems of noise and negative environmental effects. QDs are generally insoluble in alcohol and water, which has limited their applications in life sciences and biotechnology. QDs can be dispersed in water because its capping is possible with a layer of polymer. It is crucial to inhibit the non-specific binding of QDs which results in misinterpretation during explication of results obtained from experimental data. Nanotoxicity is the most fundamental issue to be addressed properly before using QDs. Optimization of size, shape, as well density of surface ROS make these QDs biocompatible for several biomedical applications [57,58,59].

#### 2.8.7. Reusability of Quantum Dot-Based FRET Sensors

This type of sensor can be designed to be reusable, but the specific design and fabrication methods used can impact their reusability. Some studies have reported successful reuse of quantum dot-based FRET sensors after proper cleaning and maintenance. For example, a study by Lim et al. (2012) demonstrated the successful reuse of a quantum dot-based FRET sensor for glucose detection, with up to 10 cycles of reuse without significant loss of sensitivity. The authors attributed the sensor’s stability and reusability to the use of a protective polymer layer to prevent the leakage of the sensing molecules and the incorporation of a glucose oxidase enzyme to improve the specificity of the sensor [60].

Other studies have reported the successful reuse of FRET sensors based on fluorescent proteins or dyes. For example, a study by Erickson et al. (2001) reported the successful reuse of a FRET sensor based on green fluorescent protein for calcium detection, with up to 10 cycles of reuse without significant loss of sensitivity. The authors attributed the sensor’s stability and reusability to the use of a genetically encoded FRET pair and the incorporation of a calcium-binding domain to modulate the FRET signal [61]. However, some FRET sensors may not be designed to be reusable or may require careful handling and storage to maintain their performance. For example, some studies have reported that FRET sensors based on organic dyes may undergo photobleaching or other forms of degradation that can limit their reusability [62].

In terms of cost and sustainability, the reusability of FRET sensors can be an important consideration. A study by Chen et al. (2022) evaluated the cost-effectiveness of FRET sensors for glucose detection and found that the use of reusable sensors significantly reduced the overall cost of the assay [63]. Additionally, the authors noted that reusable sensors could help to reduce waste and improve sustainability. Overall, the reusability of FRET sensors depends on the specific design and fabrication methods used, as well as the intended application and environmental conditions in which they will be used. With careful design and maintenance, many FRET sensors can be made to be reusable, which can help to improve their cost-effectiveness and sustainability.

#### 2.8.8. Limitations of QDs Fluorophore in FRET

In order to find the better efficiency of FRET, the size of QDs should be within 1–10 nm and also test molecule size must be less than 10 nm [58]. Here, the FRET phenomenon is very much dependent on the distance between donor and acceptor pair. Irrespectively, it is independent of the size of the molecules. In order to achieve efficient FRET, the donor and acceptor fluorophores must be close enough to each other, ideally within a distance of less than 10 nm. However, if the molecules are too large, the distance between the donor and acceptor fluorophores may be too great to allow for efficient energy transfer, resulting in a weak or undetectable signal. Additionally, larger molecules may also be more difficult to work with and may have increased background signals, leading to lower signal-to-noise ratios in FRET experiments [27].

#### 2.8.9. AIEgens Based FRET

Aggregation-induced emission fluorogens (AIEgens) can be a good source of fluorescence. Unlike traditional fluorophores, AIEgens are non-emissive in solution but become highly fluorescent when they aggregate. This unique property makes them useful for a variety of applications, including sensing, imaging, and bioimaging. AIEgens have several advantages over traditional fluorophores. They are more photostable, have higher quantum yields, and are less susceptible to photobleaching. They also have better resistance to quenching by oxygen and other chemical species. In addition to their use in fluorescence imaging and sensing, AIEgens have also been explored for use in optoelectronics, including organic light-emitting diodes (OLEDs) and organic photovoltaics (OPVs). Their unique optical properties make them promising candidates for these applications. Overall, AIEgens are a promising alternative to traditional fluorophores, and their unique properties make them useful for a variety of applications in biotechnology, materials science, and optoelectronics [64,65,66,67].

### 2.9. Role of FRET in Biosensing

FRET exhibits quantitative analysis of cellular and molecular dynamics, enzyme-kinetic reactions, and intra- and intermolecular interactions in bio-species. Biosensors based on FRET are being used to detect several bio-analytes such as viruses, bacteria, biomolecules, protein, protein kinase, peptides, genetic sequences, small-single molecule including mutation at sub-molecular as well as genetic level, etc.

**Figure 5:**
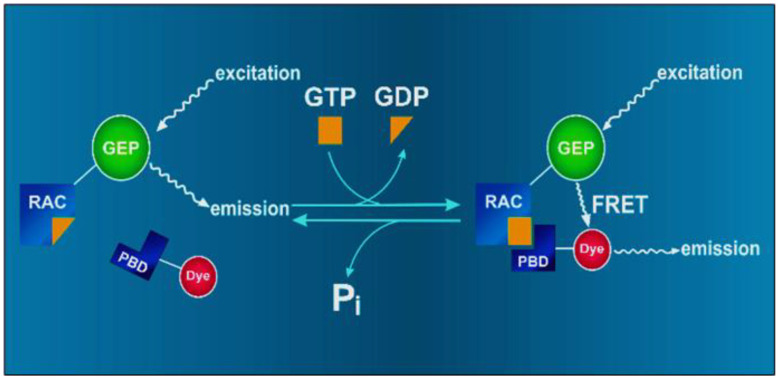
Local activity of Rac along with GTPase, which can balance protrusion and adhesions formed migratory cells by using two-chain probe-based FRET.

Figure 5 describes a fluorescent protein probed FRET biosensor. Alexa emission is rapidly differentiable from GFP molecule that shows its interaction. Probe-based FRET at different positions of single chain alters the efficiency of FRET where conformational change is observed just after interaction with particular substrate. This can be used to monitor the activation of Rac and Cdc42 [68].

FRET plays a critical role in biosensing as it allows for the detection of specific biomolecules or changes in the environment without the need for direct labeling or modification of the biomolecule. In a FRET-based biosensor, the donor and acceptor molecules are typically fluorescent proteins or small molecules that are engineered to be in close proximity to each other. When the biomolecule of interest is present, it can cause a change in the distance between both the molecules, leading to a change in the efficiency of FRET and a corresponding change in the fluorescence intensity of the acceptor. This change in fluorescence can be used to detect and quantify the biomolecule of interest. FRET-based biosensors also offer a number of advantages over other types of biosensors. For example, because FRET is a non-radiative process, it does not produce any ionizing radiation, making FRET-based biosensors safer to use and handle than other types of biosensors that rely on radioactive or ionizing radiation.

FRET-based biosensors are also highly specific, meaning that they can detect a specific biomolecule or change in the environment without being affected by other molecules or changes that might be present. This specificity is achieved by designing the biosensor to have a high binding affinity for the target biomolecule, and by engineering both molecules to be in near proximity to each other. Sensitivity of FRET biosensors is also very high, often in the picomolar range, making them able to detect very low amounts of biomolecules, which is especially important for early detection of diseases or for detecting low levels of environmental pollutants. FRET biosensors are also highly versatile, as they can detect a wide range of biomolecules and changes in the environment, and can be used in a variety of applications, such as detecting protein–protein interactions, monitoring changes in pH, or measuring the activity of enzymes [69,70,71,72,73,74]. There are some more important techniques that also play important roles in detection such as SERS (surface-enhanced Raman spectroscopy), SPR (surface plasmon resonance), and electrochemical-based sensors. In this review article, the authors are trying to compare between FRET biosensor and all the above biosensors in light of the advantages. Although FRET biosensors and SERS, SPR, and electrochemical biosensors have different advantages and disadvantages, the choice of biosensor will depend on the specific application and the requirements for sensitivity, label-free detection, multiplexing, and ease of use. SERS, SPR, and electrochemical biosensors are generally more sensitive than FRET biosensors, with detection limits in the pM or even fM range, while FRET biosensors typically have detection limits in the nM range. SERS, SPR, and electrochemical biosensors can detect analytes without the need for labels or fluorescent probes, while FRET biosensors require fluorescent labels. FRET biosensors are generally more suited for multiplexed detection of multiple analytes, as different fluorescent labels can be used for each analyte. SERS biosensors can also be used for multiplexed detection, but the number of distinguishable signals is limited by the number of available Raman peaks. However, it is not valid for the SPR and electrochemical biosensors [75,76,77].

### 2.10. Designing of FRET Biosensor

**Figure 6:**
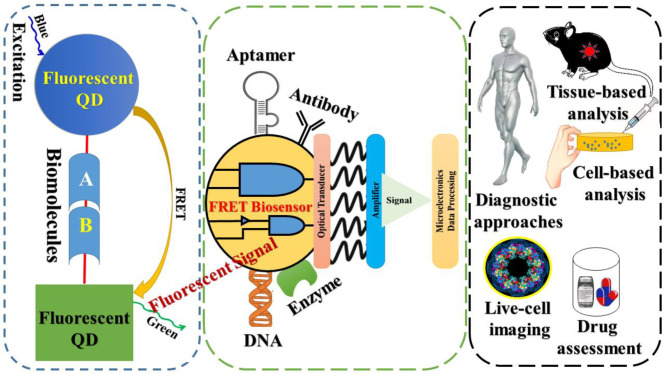
Schematic of designing and working of FRET biosensor.

The design of FRET biosensors for the detection of different molecules involves several key steps as shown in Figure 6, including the selection of appropriate fluorophores, the design of the sensor construct, and the optimization of the sensor performance. In this review article, the general guidelines have been described for designing FRET biosensors. The first and foremost step in designing FRET biosensors is selection of fluorophores. The choice of fluorophores is critical for FRET biosensor miniaturization. The donor and acceptor fluorophores should have spectral properties that allow for efficient energy transfer, and they should be stable and non-toxic. Some commonly used fluorophores for FRET biosensors include fluorescent proteins, organic dyes, and quantum dots. Further design of sensor construct is needed. The sensor construct should be designed to optimize FRET efficiency and specificity for the target molecule. This can be achieved by selecting an appropriate binding domain for the target molecule and optimizing the distance and orientation between the donor and acceptor fluorophores. The use of linkers, peptides, or other molecular scaffolds can help to fine-tune the sensor performance. Optimization of sensor performance: Once the sensor construct has been designed, it is important to optimize its performance by testing different sensor concentrations, incubation times, and experimental conditions. This can help to improve the sensitivity, dynamic range, and specificity of the sensor [78,79].

Determine the target molecule or event that the sensor will detect and select appropriate fluorophores or fluorescent proteins for the donor and acceptor. This can involve selecting fluorophores with appropriate spectral properties, such as absorption and emission spectra, and designing specific binding domains or motifs for the sensor. Clone the donor and acceptor fluorophores: Clone the DNA sequences for the donor and acceptor fluorophores into a vector that allows for expression in the target cells or organisms. This can involve using commercially available plasmids or generating custom vectors using standard cloning techniques. Design the linker: Design and clone a linker peptide or domain that will connect the donor and acceptor fluorophores together in the sensor construct. The linker can be designed to optimize the distance and orientation between the donor and acceptor molecules for maximum FRET efficiency. Some examples of commonly used linkers in FRET sensors include flexible or rigid peptides, α-helical peptides, and β-strand peptides (1). Optimize the sensor: Optimize the sensor design by adjusting the length and composition of the linker to maximize the FRET efficiency and specificity of the sensor.

This can involve testing different linker lengths, amino acid compositions, and spacer molecules to find the optimal sensor design for a given application. Express the sensor: Express the sensor construct in the target cells or organisms using transfection, electroporation, or other methods. The expression system used will depend on the specific target cells or organisms being studied. Validate the sensor: Validate the sensor by measuring its FRET efficiency and specificity using various controls and tests. This can involve using purified proteins, in vitro assays, or live cell imaging techniques to measure the FRET signal of the sensor construct and confirm its specificity for the target molecule or event. Calibrate the sensor: Calibrate the sensor by correlating the FRET signal with the concentration or activity of the target molecule or event using standard curves or other methods. This can involve using synthetic or purified standards to establish a calibration curve for the sensor, or using other methods to quantify the target molecule or event in vivo [80,81,82].

## 3. Applications of FRET Biosensor

### 3.1. Single Molecule FRET Biosensor

A single molecule FRET (smFRET) biosensor is a type of FRET-based biosensor that uses FRET to detect and quantify biomolecules at the single molecule level. In smFRET, both molecules are typically fluorescent proteins or small molecules that are engineered to be in close juxtaposition to each other. The fluorescence of the acceptor molecule is only activated when the donor and acceptor are in close proximity, so any changes in the environment that affect the distance between the two molecules will also affect the fluorescence. One of the main advantages of smFRET biosensors is that they can detect and quantify biomolecules at the single molecule level, which allows for the detection of very low concentrations of biomolecules.

Additionally, smFRET biosensors can also be used to study the dynamics of biomolecules at the single molecular level, such as protein–protein interactions or conformational changes. smFRET biosensors can be implemented in various ways, such as in solution or on surfaces. In solution, smFRET biosensors typically use a microscope to detect and track the fluorescence of individual molecules. On the other hand, surface-bound smFRET biosensors rely on the immobilization of biomolecules on a solid surface, such as a glass slide or a bead. This allows for the detection of biomolecules in a more controlled environment, and also allows for the use of more sensitive detection methods, such as total internal reflection fluorescence (TIRF) microscopy. smFRET is highly suitable for probing on various structures and finding the dynamics of biomolecules as well as detection of cancer biomarkers. This can track the biochemical reaction in a spatiotemporal-dependent way and can enlighten the transient intermediates formed during the biochemical reaction pathway which could not be accessible through the conventional method. It is one of the most promising techniques for real-time detection of the biochemical process. This can be highly useful in biomedical research for the clinical diagnosis process [83,84,85,86,87]. Small molecules, e.g., mycotoxin, pesticides, organic pollutants, peptides, small molecule hormones, drugs, veterinary drug residues, biogenic amines, and food additives, play a very important role in various biological applications such as discovery of drugs, environmental and food analysis, drug discovery, and research in physiological function and diagnosis. That is why fast detection is needed in today’s era [88,89,90,91,92].

Nowadays, several methods are used in the detection of small molecule such as chromatographic and spectroscopic approach and immunoassays [93] methods; the former methods are mainly used, but they take more time, are more complex, and are costly methods [94]. On the other hand, immunoassays methods are showing an alternative because this approach is simple and has the ability to measure high qualitative and quantitative results [95,96]. On the contrary, FRET-based biosensors play a very crucial role in manufacturing biosensors for the above small molecules which includes agri-food, pharmaceutical, clinical, and toxicological analysis. Several studies have been conducted in which FRET-based biosensors are used in the detection of cations, anions, several organic molecules, and nitric oxide [97]. Mohsin et al. (2015) developed a FRET biosensor for sensing of Zn in live cells along with microorganism [98]. Mercury ion (Hg^2+^) is a dangerous cation and it can damage the CNS (central nervous system) and endocrine system. In an experiment, Zhang et al. were able to detect Hg^2+^. In this experiment, they used boron dipyrromethene as a donor and rhodamine as an acceptor for the donor–acceptor pair of the FRET system. The use of on and off concept for adding of Hg^2+^ ion can easily show FRET occurrence by changing in intensity range [99]. As we know, cadmium ion (Cd^2+^) is harmful for health and its exposure causes calcium metabolism disorders, renal dysfunction, mutation, and itai-itai disease.

Goswami et al. developed a detection method which is FRET based, in which they used quinolone-benzothiazole as a donor and rhodamine as an acceptor with the same on and off concept used and FRET occur and help in sensing of Cd^2+^ [100]. Chung et al. developed FRET-based biosensors for the sensing of copper ion (Cu^+^), in which they used fluorescein as a donor and rhodamine moieties as an acceptor, and the use of on and off concept (intensity changes) and sensing occurred [83]. The above example was coated for cation detection. We know that anions are also important for several biological processes [79]. For example, ClO^−^ shows high reactivity towards biomolecules and also shows the property of reactive oxygen species (ROS). Zhao et al. (2014) developed FRET-based detection of ClO^−^. In this experiment, they used rhodamine/coumarin as an acceptor–donor pair for FRET occurrence. The same process of on and off led to an alternation of intensities which gave the detection result [99]. Another example is peroxynitrite (ONOO^−^), which is a reactive nitrogen species (RNS). For the detection of this ion, Qian et al. (2016) developed a FRET system. In this experiment, they used Cy3 as an energy donor and Cy5 as an acceptor [84]. Zhao et al. (2016) developed a FRET platform for the detection of sulfite (SO_3_^2−^) and bisulfite (HSO_3_^−^). They used dansyl and hemicyanine as energy donor and acceptor, respectively [85]. At the same time, Yang and colleagues developed two-photon excited fluorescence resonance energy transfer (TP-FRET). In this experiment, they used 2-acetyl-6-dialkylaminonapthalene, also known as acedan moiety [86].

### 3.2. FRET-Based Biosensor for Point-of-Need Diagnosis

FRET-based biosensors can be used for point-of-need diagnosis, which refers to diagnostic testing that can be performed at or on-site of patient care, rather than in a laboratory. It allows for rapid and accurate diagnosis, which can be especially important in remote or resource-limited settings. One example of a FRET-based biosensor for point-of-need diagnosis is a lateral flow assay (LFA). LFA is a simple, low-cost diagnostic test that can be used to detect the presence of a specific biomolecule, such as a virus or a protein marker of a disease. In a LFA, the sample (e.g., blood, urine, or saliva) is applied to a strip that is coated with a FRET-based biosensor. The biomolecule of interest, if present in the sample, will bind to the biosensor and cause a change in the fluorescence intensity of the acceptor molecule. This change in fluorescence can be visualized by a simple device, such as a handheld reader, which allows for rapid and accurate diagnosis at the point of need. Another example of a FRET-based biosensor for point-of-need diagnosis is a microfluidic device. Microfluidic devices can be used to perform multiple diagnostic tests on a single sample, and they can also be integrated with other sensors, such as optical or electrochemical sensors, to provide additional information. For example, a microfluidic FRET-based biosensor can be used to detect the presence of a particular biomolecule in blood or urine, in which the results can be read by a simple, portable device.

FRET-based biosensors are also used in portable diagnostic devices, such as handheld and wearable devices, which can be used for continuous monitoring of disease progression, treatment efficacy, and other biomarkers. In summary, FRET-based biosensors are an attractive option for point-of-need diagnosis as they are highly specific, sensitive, and versatile, and they can be integrated into simple, low-cost diagnostic devices that can be used in remote or resource-limited settings. Nowadays, the increasing demand for point-of-need diagnostics for clinical and other biomedical applications represents an urge for FRET-based biosensors that combine the bio-analytical sensing element mainly with a transducing device giving the direct result to users. This kind of device generally does not require extraordinary skills of the user. It can measure the ultra-low concentrations of analyte rapidly and has the benefit of portability. FRET-based biosensors add quantitative and significant versatility in transduction modality, which has extensively been used in biomedical research.

### 3.3. FRET Immunosensors

FRET-based immunosensors are a type of biosensor that uses FRET to detect and quantify specific proteins or other biomolecules in a sample. They are based on the principle of using an antibody or other binding molecule that specifically binds to the biomolecule of interest, and then using FRET to detect the binding event. Immunosensors are the device that senses or detects the immune reaction between antigen and antibody. Immunosensors are one of the large family of biosensors in which a highly selective and sensitive receptor layer is present which has immobilized bio components such as antibodies, antigen, hapten, etc., as an immunological receptor for the detection of immunosensitive analyte. In this type of sensor, the receptor layer contacts directly to the transducer layer on which biomolecular interaction is converted and transformed into a measurable amount of signal. FRET-based system is an important platform for the immune molecules detection that is can simply say that immunosensor [87,101].

FRET-based immunosensors can be used in a lot of applications, such as the detection of disease markers, toxins, or pathogens in clinical or environmental samples. They can also be used for the quantification of proteins in complex biological mixtures, such as cell lysates or blood serum. One example of a FRET-based immunosensor is a sandwich assay, where a capture antibody is immobilized on a solid surface, and a sample containing the biomolecule of interest is added. The biomolecule will bind to the capture antibody and a labeled detection antibody, which is conjugated with a FRET donor and acceptor, is added. The FRET signal is measured and used to quantify the amount of biomolecule in the sample. Another example of a FRET-based immunosensor is a competitive assay, where a labeled biomolecule is added to the sample, and the FRET signal is measured to quantify the amount of biomolecule that is bound to the binding molecule. FRET-based immunosensors are a powerful tool for detection and quantification of biomolecules, as they offer high sensitivity, specificity, and selectivity, which make them an important tool for diagnosis, surveillance, and monitoring of diseases, or for the detection of environmental contaminants.

FRET immunosensors are also being used in combination with other techniques such as microfluidics and lab-on-a-chip to make them more portable, cost-effective, and faster. There are several detection methods that have been discovered and studied [102]. In this review, we are trying to focus on the FRET-based immune platform. From many experiments, it is found that FRET immunosensors are mainly linked by –SiOCN/-NH_2_, -COOH/-NH_2_, and biotin/strepatividin [103,104]. There are several immunosensors that have been developed to date such as fluorescent sandwich immunoassays, standard enzyme links, etc., but compared to these methods, FRET-based immunosensors show unprecedented character, e.g., separation step, phase of washing (free), great stability, uniqueness, and ratiometric measurement [105,106]. As we know, there are a lot of studies that have been conducted in the field of FRET-based biosensors. Most of them are successful [107,108]. In 2011, Kattke and his group developed a successful FRET-based biosensor for sensing mold-spores that are present in solution, in which they performed conjugation of quantum dots (QDs) which must be fluorescent to antibodies. Then, these QDs were blended with mold-spores solution with a labeled quenching agent. It is found that when unlabeled spores are mixed with solutions, then fluorescence recovers back. In this experiment, spores detection limit was 103 spores per milliliter [109].

Stringer et al. (2008) successfully developed a FRET-based immunosensor for antigen sensing by changing its conformational structure when antigen conjugated with antibody. In this experiment, they made conjugation of protein with QDs and this conjugation helps in orientation of an attachment of labeled dye IgG-antibody; contrary Troponin I protein and FRET occurred. Its LOD is 200 nM. Xu et al. (2014) developed a FRET immunosensor for sensing of aflatoxin-B_1_, also called AB_1_. They used two different types of QDs: monovalent monoclonal antibody (mAb labeled QDs) as a donor and multivalent hapten (labeled green QDs) as an acceptor. These two types of QDs were used to prevent coagulation. FRET occurs between two QDs and it is valid even for the detection of 0.19–16 pM, which is why it is the best candidate for the detection of AFB_1_ at very low concentration [107].

Pan et al. (2022) developed a FRET immunosensor for the rapid sensing of dicofol, which are bimetallic nanoclusters. In this experiment, firstly, they made gold-silver bimetallic nanocrystals (Ag-Au NCs) and gold nanofibers (AuNFs), which were both in liquid and PADs, which are paper-based analytical devices. After that, they conjugated Au-Ag NCs/antigen and Au-Ag nanocrystal as a donor, whereas they conjugated Au-NFs/antibody as an acceptor. Overlapping occurred and FRET developed. When DICO mixed with antibody conjugation, then FRET inhibited; it takes a small amount of time to detect DICO in liquid as well as PADs. This experiment was successfully performed in three types of tea sample. This research opened many opportunities regarding on-site detection method [106,110].

Sruti et al. developed a FRET immunosensor for *Helicobacter pylori* detection (a causative agent gastric carcinoma). In this experiment, they synthesized carbon dots (functionalized) and conjugated it with *H. pylori* antibody, which works as fluorescent prob (i.e., FCDs/Ab). When GO, i.e., graphene oxide, was mixed with FCDs/Ab, quenching occurred. Moreover, when *H. pylori* was mixed, the distance between FCD/Ab and GO changed. It was due to interaction of Ab and *H. pylori* with LOD of 10 cells/mL [111]. In the same year, Kseniya et al. developed an immunosensor for rapid detection of ochratoxin A, also called OTA (a toxic contaminant of foods product). In this experiment, they used amino methyl fluorescein (AMF), also called fluorophore, as a donor and gold nanoparticles as an acceptor. It was found that OTA–AMF showed the fluorescence when competition reaction occurred between OTA and AMF, and free OTA bonding with OTA antibodies, which were labeled with gold nanoparticles, resulted in a relation between OTA–AMF fluorescence and concentration of OTA (0.09 to 3.12 ng mL^−1^) with LOD of 0.02 ng mL^−1^ [112].

Table 1 shows the year-wise development of FRET-based immunosensors with respect to donor, acceptor, LOD, and CR.

### 3.4. Enzymatic FRET Biosensor

Enzymes are the biocatalysts which enhance the rate of biochemical reaction by lowering the energy of activation. They are involved in many important biological processes such as metabolism, signal transduction, and DNA replication. Enzyme-based biosensors utilize the catalytic activity and binding affinity of enzyme with analyte, which we targeted for the detection. An enzymatic FRET biosensor is a type of biosensor that uses FRET to detect the activity of enzymes in a sample. In an enzymatic FRET biosensor, the enzyme of interest is typically immobilized on a solid support, such as a bead, a film, or a substrate surface. A FRET-labeled substrate is then added to the sample and the enzyme catalyzes the conversion of the substrate into a product. The FRET signal is measured and used to quantify the enzyme activity. One example of an enzymatic FRET biosensor is a glucose biosensor, which uses the enzyme glucose oxidase (GOx) to detect glucose in a sample. The GOx catalyzes the glucose oxidation into gluconic acid and hydrogen peroxide. In this case, a FRET-labeled hydrogen peroxide substrate is added, and the FRET signal is measured and used to quantify the glucose concentration. Another example of an enzymatic FRET biosensor is a kinase biosensor, which uses the enzymes kinase to detect the presence of phosphorylated proteins and also to study their activity. Kinase catalyzes phosphate group transfer from ATP to a protein, resulting in a change in the protein’s activity.

There have been a lot of studies conducted on the enzymatic FRET-based biosensor, whether in vivo or in vitro [70,121,122]. This type of biosensor is mainly manufactured for the detection of protein with the concept of two kinds of molecules, i.e., donor molecules and acceptor molecules, and unique recognition module for the targets [123,124]. Conformational changes occurred by an on and off process of FRET. In 2017, Greta et al. detected neutrophil elastase (NE) activity by devising a FRET biosensor. In this experiment, they used YFP, i.e., yellowish green Aequorea fluorescent protein (a variant of green fluorescent protein (GFP) which is a bioluminescent polypeptide protein that is extracted from jellyfish *Aequorea victoria*), and CFP, i.e., cyan Aequorea fluorescent proteins (the color variant of CFP). YFP is used as an acceptor and CFP is used as a donor. Firstly, YFP was combined with CFP via a covalent bond that had NE recognition sequence showing FRET sensitivity [125].

Ghosh et al. detected protein by devising a biosensor in which they used synthesized pyrimidine derivatives as fluorescent probe; adding this probe, i.e., ANHP, shows the conformational changes of protein and develops high sensitivity. Sidhu et al. detected thioredoxin reductase (TrxR) activity in cancer cells by the use of selective biotin/CDs/naphthalimide with LOD of 7.2 × 10^−8^ TrxR [126]. With the same concept, Lee et al. designed an enzymatic printed inkjet FRET biosensor. In this experiment, they used 5-FAM and QXL520 as a donor–acceptor pair [122]. Qian et al. devised a sensor for sensing the activity of intracellular telomerase by use of switchable FRET system. Several research studies have been conducted on the basis of FRET biosensors such as detection of caspase-3, reductase, protease collagenase (MMP-9, MMP-2), and other zymo-proteins present in live cell [127]. Xu et al. developed a double-labeled FRET sensor for imaging of MMP-2 and MMP-7 of living cells performed in a single excited wavelength [128]. Li also fabricated a dual FRET sensor for the detection of both MMP-7 and MMP-2 for the monitoring of cancerous drug and observing the therapeutic behavior [129]. In this experiment, they used FAM, also called carboxyfluorescein, as a donor and two dabcyl molecules as an acceptor. An improved FRET-based biosensor was also developed by Limaskul for screening the enzymatic activities on the surface of cells. Enzymatic FRET biosensors are a powerful tool for the detection and quantification of enzymatic samples, as they offer high sensitivity, specificity, and selectivity. They are used in a variety of applications such as in medical diagnostics, environmental monitoring, and drug discovery. Enzymatic FRET biosensors also have the potential for in vivo applications, where the enzymes are immobilized on nanoparticles and delivered inside the body to detect and quantify specific enzymes at the site of interest.

Table 2 shows the year-wise development of FRET-based enzymatic biosensors with respect to donor, acceptor, LOD, and CR.

### 3.5. Aptamer Based FRET Biosensor 

Aptamers are nothing but a short stretch of oligonucleotides such as DNA or RNA or any peptide molecules which can bind with its target with very high affinity as well as specificity. It is a category of nucleic acid ligands with high affinity that are selected through ssRNA/ssDNA binding with a highly specific target molecule from the region of library in vitro [134]. Aptamer shows the specific combination properties, hence resulting in high stability, high selectivity, high affinity, high thermal stability, and low cost [135]. They are stable and non-toxic, having a lack of immunogenicity, rapid tissue penetration, and the ability to be easily modified with different functions for labeling. As we know, various proteins cause diseases such as heart-, kidney-, bone-, eye-, and brain-related diseases. Aptamer catches these target proteins and processes them further to make an effective drug. Aptamer has been regarded as an alternative to antibodies because it has several advantages over antibodies.

In 1989, with the discovery of aptamers, the biosensing community immediately recognized its importance. Since aptamers are highly selective and specific, they can be used to detect unlimited kinds of analytes. Currently, mass sensitive and optical aptasensors are mostly used. This type of biosensor can also be called a nucleic acid-based FRET biosensor (Chen et al. 2014). This combination of FRET biosensor may be sandwich, non-specific, and with displacement assemblies [136,137,138]. In 2017, Auer et al. designed a rapid and free background-based FRET biosensor for DNA-PAINT, i.e., DNA point accumulation in nanoscale topography. This gives very rapid results with super resolution image of DNA and great quality [136]. In 2018, Japsen and group developed a FRET-based biosensor for RNA tracking in *E. coli*. In this experiment, they used RNA aptamers to develop a reversible and dynamic RNA nanodevice. In 2017, Tian et al. developed an aptasensor for sensing ochratoxin A, also called OTA, in food chains system. In this experiment, they used cerium oxide nanoparticles (colloidal) as a donor and graphene quantum dots, also called GQDs, as an acceptor. In this experiment, they showed that FRET occurred between the donor and acceptor in the absence of OTA aptamer, and in presence of it, no FRET occurred. Moreover, in the presence of OTA, FRET occurred that showed a detection and high selectivity towards OTA with LOD of 2.5 pg/mL. In the next year, Wu et al. devised an aptamer-related FRET biosensor to detect hybridization of DNA by using UCNP, i.e., up-conversion nanoparticle, and stain of nucleic acid which is a specific intercalator of dsDNA (SYBER Green I or SG) [137]. In the same year, Xu designed a biosensor for the sensing of chiral nanorods (dimer) based on self-assembly of miRNA directed intracellular [139].

Table 3 shows the year-wise development of aptamer-based FRET biosensors with respect to donor, acceptor, LOD, and CR.

### 3.6. Tissue Based FRET Biosensor

Tissue-based FRET biosensors are a type of biosensor that uses FRET to detect and quantify specific biomolecules in a tissue sample. They are based on the principle of using a FRET-labeled probe that particularly binds to the biomolecule of interest in the tissue and then using FRET to detect the binding event. Tissue-based FRET biosensors can be used in a variety of applications such as in medical diagnostics, drug discovery, and basic research. They can be used to detect and quantify specific proteins, nucleic acids, or other biomolecules in tissue samples, such as tumors, healthy tissue, or cells. It can also be used to sense and measure drug molecules, hormones and their toxins, etc. This type of FRET biosensor is potentially used in various fields of biomedical research such as pharmacology, physiology, biodefense, etc. Mainly tissue-based FRET biosensors may be devised from genetically modified cells or modification at the genetic level to introduce the biosensor fluorescent protein in the tissue of animals.

Bio-photonics has been providing the most versatile basis for tissue-based FRET biosensors in which the light output from biosensor cells can be obtained in the form of fluorescence or bioluminescence. Tissue-based FRET biosensor utilizes the protein–protein interactions to detect any molecule, i.e., fusion protein may be used to produce resonance energy transfer. One example of a tissue-based FRET biosensor is a FRET-based in vivo imaging, where a FRET-labeled probe is administered to an animal model and the FRET signal is used to detect and quantify specific biomolecules in vivo. This can be used to study the distribution, activity, and expression of biomolecules in different tissues and organs, and also to monitor the progression of diseases. Another example of a tissue-based FRET biosensor is a FRET-based tissue microarray, where small tissue samples are arrayed on a slide and a FRET-labeled probe is used to detect and quantify specific biomolecules in the samples. This can be used to study the expression of biomolecules in different tissue samples and also to compare the expression of biomolecules in different disease states.

Tissue-based FRET biosensors are a powerful tool for the detection and quantification of specific biomolecules in tissue samples, as they offer high sensitivity, specificity, and selectivity. They can provide important information about the distribution, activity, and expression of biomolecules in tissues, which can be used to understand the underlying biological processes and to identify new targets for therapeutic intervention. There are various experiments that have been conducted in the field of tissue-based FRET biosensors since 2008. Bouchala et al. (2016) designed a biosensor in which they used 100 nm size of fluorescent nano-emulsion droplets and it encapsulated lipophilic near infrared cyanin with the help of phenylborate for in vivo imaging of mice [143]. In this experiment, they found that FRET nanocrystals in blood circulation are preserved at 93% for 6 h but it drops to 66% in the liver. In another experiment, Chen et al. developed a FRET biosensor, an acid active tumor targeting nanoplatform 2,3-dimethylmaleic anhydride (DA), and a trans-activating transcriptional activator, also called TAT polycaprolactone. It will prevent the interaction of TAT in the bloodstream. Guo et al. (2018) devised a FRET biosensor and observed signal of FRET between the camptothecin and maleimide thioether bond and also tracked the drug releasing process [144].

### 3.7. FRET-Based pH Sensor

FRET-based pH sensors are biosensors that use FRET to detect changes in pH. They work by using a FRET-labeled probe that changes its FRET efficiency when the pH changes, allowing for the detection and quantification of pH changes. Acid–base balance is the main key to regulate the biological process and cells must have certain range of pH. Otherwise, unstable pH may lead to various diseases. Hence, it becomes necessary to devise the biosensor which can monitor the intracellular pH to monitor the physiological process. Fluorescent probes are the choice of research scholars all across the globe as a pH detection tool as they are simple, fast, and reliable.

The first colorimetric sensor was devised using rhodamine (Rh-TPE) to monitor change in pH using FRET as it is highly sensitive having excellent cell permeability and minimal toxicity. FRET-based pH sensors typically consist of a FRET donor and acceptor, which are typically fluorescent dyes that are attached to a pH-sensitive molecule. There are different types of FRET-based pH sensors; some are based on the use of pH-sensitive peptides, others use pH-sensitive proteins, and others use pH-sensitive organic molecules. FRET-based pH sensors have many applications such as in medical diagnostics, environmental monitoring, and basic research. They can be used to detect pH changes in biological samples such as blood, saliva, urine, or in cells and tissues and to monitor the pH changes that occur during disease progression or during the response to drugs or other therapies. FRET-based pH sensors are also used in industry to monitor pH changes in food and beverage production or in water treatment.

FRET-based pH sensors have many advantages over traditional pH sensors such as high sensitivity, specificity, and the ability to detect pH changes in complex samples. They also offer the possibility of real-time monitoring of pH changes in vivo using fluorescence imaging techniques which can provide important information about the underlying biological processes and to identify new targets for therapeutic intervention. FRET-based biosensors can be used to detect and quantify hard water ions such as calcium and magnesium in a water sample. The principle of these biosensors is based on using a FRET-labeled probe that specifically binds to the hard water ions of interest, and then using FRET to detect the binding event. One example of a FRET-based biosensor for hard water ions is a sensor that uses a FRET-labeled peptide that binds to calcium ions. The FRET signal generated by the binding of the peptide to calcium ions can be used to detect and quantify the presence of calcium ions in a water sample. Another example is a biosensor that uses a FRET-labeled protein that specifically binds to magnesium ions, where the FRET signal is used to detect and quantify the presence of magnesium ions in a water sample. FRET-based biosensors for hard water ions are sensitive and specific and can be used in a variety of applications such as in environmental monitoring, water treatment, and industrial processes. They can be used to detect and quantify hard water ions in drinking water, industrial water, or in water used in food and beverage production.

FRET-based biosensors can also be used to monitor the efficiency of water treatment processes such as ion exchange, reverse osmosis, or demineralization by detecting the presence of hard water ions in the treated water. FRET-based biosensors are a powerful tool for the detection and quantification of hard water ions in water samples as they offer high sensitivity, specificity, and selectivity. They can provide important information about the presence of hard water ions in water samples, which can be used to understand the underlying water chemistry and to optimize water treatment processes. As we know, from various studies that hard water contains more minerals than soft water, we can say that in hard water, concentration of cations is more than +1. Ca and Mg ions are mainly present in hard water. Dibyendu et al. (2013) developed a FRET-based biosensor for the detection of hard water sample in which they used acriflavine, also called Acf, and rhodamine B (also known as RhB) as donor and acceptor pair. Both Acf and RhB show the highly fluorescent activity [145]. In an experiment, Sahara et al. showed that FRET develops in a mixture of both the dyes [146]. As it is obvious that FRET is affected when the distance between donor and acceptor changes or is altered by the introduction of external agencies in the mixture, it is also affected by the changes in microenvironment of the mixture. From many experiments, it has also been found that if the distance between the donor and acceptor decreases, then FRET increases, and if the distance between donor and acceptor increases, then FRET decreases [147]. In this study, they opted Ca^2+^ and Mg^2+^ because their concentration is high in hard water. When the concentration of these ions increases, then it is found that FRET increases. By this simple process, researchers are able to find the sensing of hard water by the use of FRET concept between AcF and RhB [145]. Arsenic (V), i.e., As^+5^, is also detected by FRET-based biosensor in water sample by the above same dye with LOD of 10 µgL^−1^. Heavy metal ions such as Hg^+2^ are also found in the water sample which makes it hard and causes health problems; hence, its detection is also important [148]. For this study, Jaba et al. developed a FRET-based sensor for the detection of Hg^+2^ [149]. In this same work, the researchers used N, N-dioctadecyl thiacyanine perchlorate, i.e., NK, as a donor and octadecyl rhodamine B chloride, i.e., RhB, as an acceptor. Firstly, they introduced both molecules in thin film using Langmuir–Blodgett (LB) and in spin coating (SC), and after that, both were dipped into Hg^+2^ solution, i.e., sample solution. After performing spectroscopic analysis, they found detection of Hg^+2^ with detection limit of 9.13 ppb in LB and 11.7 in SC films [149].

### 3.8. FRET-Based Ion Sensor

FRET-based ion sensors are biosensors that use FRET to detect and quantify specific ions in a sample. They work by using a FRET-labeled probe that particularly binds to the ion of interest, and then using FRET to detect the binding event. There are different types of FRET-based ion sensors depending on the ion of interest. For example, a FRET-based calcium sensor would use a FRET-labeled probe that specifically binds to calcium ions, while a FRET-based magnesium sensor would use a FRET-labeled probe that specifically binds to magnesium ions. FRET-based ion sensors can be based on a variety of FRET-labeled probes such as peptides, proteins, or organic molecules. The FRET-labeled probes are designed to specifically bind to the ion of interest and the FRET signal generated by the binding event can be used to detect and quantify the presence of the ion in the sample. FRET-based ion sensors have many applications such as in medical diagnostics, environmental monitoring, and basic research. They can be used to detect and quantify ions in biological samples such as blood, saliva, urine, or in cells and tissues. They can also be used to monitor the efficiency of ion exchange processes in water treatment or to detect and quantify ions in industrial processes.

FRET-based ion sensors have many advantages over traditional ion sensors such as high sensitivity, specificity, and selectivity. They can provide important information about the presence of specific ions in a sample, which can be used to understand underlying chemical processes and to optimize industrial processes. Additionally, they can be used in real-time monitoring of ions in vivo using fluorescence imaging techniques, which can provide important information about the underlying biological processes and to identify new targets for therapeutic intervention. Several metals influence the living cell directly or indirectly. It is needed in very trace amount to regulate the biological reaction inside the cell or body. Higher dose may be hazardous for the health, as even metal toxicity such as nickel, arsenic, and lead may cause chronic toxic effects on human health. To monitor the uptake and metabolism of silver ions in vivo as well as in vitro, the FRET-based nanosensors may be used which can be made by using periplasmic protein CusF, a part of the CusCFBA efflux complex that is involved in developing resistance against metal ions such as copper and silver in copper and *E. coli*. This FRET-based nanosensor was devised by the combination of two fluorescent proteins (donor and acceptor) at the N terminal and C terminal of silver-binding protein (CusF).

### 3.9. FRET-Based Hard Water Sensor

Sensing of hardness of water by evaluating the concentration of calcium and magnesium present in water can be monitored using a FRET-based sensor which utilizes the principle of FRET in which the FRET efficiency between two laser dyes, acriflavine (Acf) and rhodamine B (RhB), changes in the presence of permanent hard water having CaCl_2_ and MgCl_2_.

### 3.10. FRET-Based Wearable Biosensor

FRET-based wearable biosensor is a type of device that uses the principle of FRET to measure biological signals such as glucose levels or pH in a non-invasive manner. The device typically consists of a FRET-based sensor that is integrated into a wearable device such as a patch or a wristband which allows for continuous monitoring of the biological signal. FRET-based biosensors can potentially be used for numerous applications including diabetes management, monitoring of pH levels in the body, and detecting certain types of cancer.

### 3.11. Other FRET-Based Sensor

Apart from the above discussed FRET-based sensors, there are several other sensors that can be used in living cell mechanopharmacological, mechanotransduction imaging and drug screening [142,143,144,145,146,147,148,149,150]. We know that FRET-based biosensor depends on distance within donor–acceptor pair, but multi-FRET biosensor works independent of distance. Saha et al. (2016) developed a multi-FRET biosensor system to be used for detection at the molecular level by the use of three different dyes namely, acriflavine (Acf), rhodamine B (RhB), and pyrene (Py). In this experiment, they found that energy is transferring from pyrene to rhodamine via qcriflavine. This multi-phase FRET helps in studying interaction at the molecular level [144]. Xu (2017) designed a molecular imprinted fluorescent biosensor for the detection of doxorubicin with LOD of 13.8 nM [150]. Here, doxorubicin functioned as a template. From various studies, we can say that the FRET concept has also been used in thermal biosensors [151,152,153,154]. Huang et al. (2017) devised a FRET-based biosensor for sensing the polymeric micelles in hydrogel and also monitoring the release mechanism of micelles. In this experiment, they used FITC and rhodamine B as donor–acceptor pair. Firstly, the researchers made a PECT triblock copolymer and conjugated it separate with donor and acceptor such as FITC/PECT and PECT/RB and co-assembled both of them within 1–10 nm. It was found that at 465 nm excitation, it generated stronger emission at 590 nm wavelength. Now, hydrogel is injected into mice to determine the delivery and degradation of micelles via FRET imaging [155].

There have been several recent advances in FRET-based sensors including the development of new FRET-labeled probes, improved detection methods, and the integration of FRET-based sensors into microdevices and biosensors. One recent advance has been the development of new FRET-labeled probes, such as fluorescent proteins, nanoparticles, and organic dyes. These probes have improved brightness, stability, and specificity, allowing for more sensitive and selective detection of the target analyte. Another recent advance has been the development of new detection methods such as fluorescence lifetime imaging and single-molecule FRET which have improved the sensitivity and specificity of FRET-based sensors. Recent advances in micro fabrication and nanotechnology have also enabled the integration of FRET-based sensors into microdevices and biosensors such as lab-on-a-chip devices and portable biosensors. These devices can be used in a variety of applications such as medical diagnostics, environmental monitoring, and industrial processes.

In addition, FRET-based biosensors are also being used in combination with other techniques such as SPR, i.e., surface plasmon resonance, to enhance selectivity and sensitivity detection. Professor Lambertus Hesselink worked on C-aperture, which has amazing plasmonic properties. One interesting property of C-apertures is that they can exhibit strong circular dichroism, which is the differential absorption of left-handed and right-handed circularly polarized light. This property arises from the asymmetric shape of the C-aperture, which breaks the symmetry of the plasmonic response [156]. Nano C-apertures have been used as a platform for FRET measurements due to their unique plasmonic properties. When two fluorophores are placed in close proximity to a C-aperture, the plasmonic resonance of the aperture can enhance the FRET efficiency by increasing the interaction between the donor and acceptor fluorophores. The working principle of nano C-apertures involves the interaction of light with the nanoscale aperture and the surrounding metal film. When light is incident on the aperture, it creates surface plasmons, which are collective oscillations of electrons in the metal film. The plasmons can enhance the local electromagnetic field, leading to increased excitation and emission rates of nearby fluorophores. Nano C-apertures can be fabricated using techniques such as electron beam lithography or focused ion beam milling. The size and shape of the aperture can be tuned to control the plasmonic properties and optimize the FRET efficiency.

FRET-based biosensors are also being used to detect disease markers in blood and other biological samples such as cancer biomarkers and to detect and quantify small molecules such as neurotransmitters and hormones.

Table 4 shows the year-wise development of several FRET-based biosensors such as tissue-based biosensor, thermal biosensor, and other FRET sensors with respect to donor, acceptor, LOD, and CR.

## 4. AI, ML and IOT in FRET-Based Biosensor: Future Prospective

### 4.1. AI and ML in FRET-Based Biosensor

Artificial intelligence (AI) and machine learning (ML) have been increasingly used in FRET-based biosensors in recent years to improve the sensitivity, specificity, and selectivity of detection. One way that AI has been used in FRET-based biosensors is through the development of machine learning algorithms that can analyze the FRET signals generated by the biosensor. These algorithms can be trained to recognize patterns in the FRET signals that are indicative of the presence of a specific analyte and can be used to detect and quantify the analyte in a sample. Another way that AI has been used in FRET-based biosensors is through the development of virtual screening methods that can predict the binding affinity of potential FRET-labeled probes for a specific analyte. These methods can be used to rapidly identify potential probes for a specific application, reducing the time and cost of biosensor development. In addition, AI algorithms such as deep learning have been used to analyze large datasets of FRET signals, which can be used to improve the accuracy of the FRET-based biosensors, and also to identify new binding sites or new binding partners. AI-based techniques can also be used to improve the design of FRET-based biosensors, such as optimizing the probe sequences, the FRET pair, and the assay conditions, to improve the sensitivity and selectivity of the biosensor. Overall, the integration of AI into FRET-based biosensors has the ability to improve the performance of these biosensors, making them more effective for numerous applications such as disease diagnosis, industrial process control, and environmental monitoring [158]. In a study published in the journal “ACS Sensors” in 2019, researchers developed a FRET-based biosensor for detecting the neurotransmitter dopamine in the brain. They used a machine learning (ML) algorithm to optimize the design of the biosensor and improve its sensitivity. The researchers used a FRET pair consisting of a blue fluorescent protein (mTurquoise2) and a yellow fluorescent protein (cp173Venus) to detect changes in dopamine levels in the brain. They then used an ML algorithm to analyze the data generated by the biosensor and identify the optimal conditions for measuring dopamine levels. The ML algorithm was trained on a dataset of FRET signals from different dopamine concentrations, and it was able to learn the relationship between the FRET signal and dopamine concentration. The algorithm then used this knowledge to predict the optimal FRET ratio for detecting dopamine in real time. The optimized FRET-based biosensor was able to detect dopamine with high sensitivity and selectivity, and it was successfully used to monitor dopamine release in the brains of living mice. The researchers suggest that this approach could be applied to other FRET-based biosensors for detecting a wide range of analytes.

### 4.2. IoT for FRET-Based Biosensor

The Internet of Things (IoT) has the potential to greatly enhance the capabilities of FRET-based biosensors by connecting them to a network of devices and enabling real-time monitoring and analysis of the data generated by the biosensors. One way that IoT can be used with FRET-based biosensors is through the integration of wireless communication technologies, such as Bluetooth or Wi-Fi, which can allow the biosensors to transmit data to a central hub or cloud-based platform for real-time analysis. This can enable remote monitoring of the biosensors and real-time data analysis, which can be useful for applications such as industrial process control, environmental monitoring, and medical diagnostics. IoT can be used with FRET-based biosensors through the integration of microfabrication and nanotechnology, which can enable the development of small portable biosensors that can be easily connected to the IoT. These devices can be used in a variety of applications such as medical diagnostics, environmental monitoring, and industrial processes. IoT-enabled FRET-based biosensors can also be connected to other devices, such as smartphones, tablets, or computers, which can enable real-time data visualization, analysis, and notifications. Additionally, IoT can be integrated with AI algorithms such as machine learning to analyze the large datasets generated by the FRET-based biosensors, which can be used to improve the accuracy of the biosensors and to identify new binding sites or new binding partners. Overall, the integration of IoT into FRET-based biosensors has the potential to greatly enhance the capabilities of these biosensors and to make them more effective for a wide range of applications. FRET is a widely used photo-physio-chemical technique which works in a distance-dependent manner and works even at ultra-low concentration; thus, it will be the spectroscopic ruler over the other conventional techniques. FRET-based biosensors will be highly advantageous in the future as they can monitor the interaction at ultra-low concentrations, even at picomolar and nanomolar concentrations.

### 4.3. Recent Challenges

There are several challenges that have been identified in the development and use of FRET-based biosensors. Low signal-to-noise ratio is one of the major challenges in FRET-based biosensors, which can make it difficult to detect and quantify small changes in FRET signal. Development of FRET-labeled probes with high specificity for a particular target is a challenging task. The specificity of the probes is crucial for accurate detection and quantification of the target. FRET-based biosensors can be affected by environmental factors such as temperature, pH, and ionic strength, which can affect the binding kinetics of the probes and the FRET signal. FRET-based biosensors can also be affected by interference from other substances in the sample such as auto fluorescent or quenching agents, which can affect the accuracy of the measurements. FRET-based biosensors are often used in complex matrices such as blood, urine, or tissue, which can contain a variety of other biomolecules that can interfere with the FRET signal.

Developing a FRET-based biosensor can be expensive and time-consuming, involving synthesizing, labeling, and functionalization of probes, which can be a challenge for some applications. FRET-based biosensors generate large datasets that require specialized software and computational resources for analysis, which can be a challenge for researchers. FRET-based biosensors are typically designed to detect a single analyte, but many applications require the detection of multiple analytes, i.e., multi-parametric sensing, which again is a challenging task. Overall, these challenges highlight the need for continued research and development in FRET-based biosensors to improve the sensitivity, specificity, and selectivity of detection and to make them more effective for a wide range of applications.

FRET-based biosensors have become increasingly popular in recent years for studying various biological processes. However, there are still some challenges that need to be addressed in order to improve the performance and reliability of FRET-based biosensors. One of the major challenges in FRET-based biosensors is the low signal-to-noise ratio, which can lead to false positive or negative results. This can be due to factors such as background fluorescence, autofluorescence, and photobleaching. FRET-based biosensors often require high specificity in order to accurately detect and measure the concentration of the target molecule. However, many biosensors can also detect related molecules, leading to cross-reactivity and potential false positives. Another challenge of FRET-based biosensors is the limited dynamic range, which can limit the ability to accurately detect changes in the concentration of the target molecule. This can be particularly problematic when studying biological processes that involve rapid changes in concentration. FRET-based biosensors often require the use of high-intensity light, which can be phototoxic to living cells and tissues. This can lead to cell death, tissue damage, and other unintended effects. FRET-based biosensors can be prone to degradation and instability over time, which can lead to changes in their performance and reliability.

FRET-based biosensors have many advantages, and addressing these challenges will be important in order to improve their performance and broaden their range of applications in biological research. FRET efficiency: The FRET efficiency is dependent on the distance between the donor and acceptor fluorophores. However, changes in the local environment, such as pH or ionic strength, can affect this distance and thus alter the FRET efficiency. This can lead to inaccuracies in the measurement of the target molecule concentration. Photobleaching: FRET-based biosensors are prone to photobleaching, which can result in a loss of signal intensity over time. This can make it difficult to measure the target molecule concentration accurately, particularly during long-term experiments. Cell permeability: FRET-based biosensors may not be able to penetrate the cell membrane or be efficiently internalized by cells, making it difficult to measure target molecules inside living cells. This can limit the range of biological processes that can be studied using FRET-based biosensors. Sensor design: The design of FRET-based biosensors can impact their performance, sensitivity, and specificity. Improving the design of these biosensors, such as optimizing the choice of fluorophores or changing the linker length between the donor and acceptor, can help overcome some of the challenges associated with these biosensors. Quantifying FRET-based biosensors can be challenging, particularly when using microscopy techniques. This can be due to difficulties in accurately measuring fluorescence intensity, as well as potential variability between different experimental setups. Standardization and optimization of quantification methods will be important for improving the accuracy and reliability of FRET-based biosensors.

## 5. Conclusions

Since the past few decades, FRET-based biosensors have been helpful for the research community to monitor inter- or intramolecular interaction, but in the modern edge, we can consider FRET as the most powerful tool which has been used extensively in devising biosensors and nano-biosensors used in various fields such as biology, chemistry, nanomedicine, nanotechnology, and nanobiotechnology. It allows for sensitive, real-time monitoring of biological processes and molecules by detecting changes in fluorescence. FRET biosensors/nano-biosensors can be designed to detect specific targets, making them highly sensitive, selective, and specific.

Like any other technology, FRET sensors have some limitations that need to be addressed very well. FRET sensors have a limited spatial resolution, which can make it difficult to visualize and measure biological events occurring at the nanoscale or subcellular level. FRET sensors can suffer from non-specific binding, which can lead to background signal and false-positive results. Non-specific binding can be caused by factors such as electrostatic interactions, hydrophobic interactions, and steric hindrance. While FRET sensors are generally sensitive, they may not be sensitive enough to detect low concentrations of the target molecule or event. Improving the sensitivity of FRET sensors may require optimizing the sensor design, using more sensitive fluorophores such as quantum dots, or increasing the excitation light intensity. FRET sensors may also suffer from cross-reactivity with related molecules, leading to false-positive results. Cross-reactivity can be minimized by optimizing the sensor design and selecting specific binding domains or motifs. As mentioned earlier, FRET sensors often require high-intensity light, which can be phototoxic to living cells and tissues. This can limit the applicability of FRET sensors in certain biological contexts, such as studying long-term cellular responses. FRET sensors require calibration to convert the fluorescence signal into a quantitative measurement of the target molecule or event. However, the calibration process can be time-consuming and may not be applicable in certain biological systems or environments.

With the advancement of FRET technology and its increasing use in various applications, it is expected to play a significant role in improving our understanding of biological systems and advancing medical diagnostics. This technology has changed the way of designing biosensors. The application of FRET-based biosensors has greatly expanded in recent years due to their advantages in detecting biological molecules and processes with high sensitivity, selectivity, and specificity. FRET biosensors have been applied in various fields, including neuroscience, drug discovery, cancer research, and medical diagnostics. The use of FRET biosensors has led to a better understanding of biological systems and the development of new treatments and diagnostic tools. As technology continues to advance, it is expected that the applications of FRET-based biosensors will continue to increase and play a significant role in improving human health and well-being. The integration of AI and machine learning (ML) in FRET-based biosensors has the potential to revolutionize the field of biosensing. The application of AI and ML algorithms can improve the accuracy and efficiency of FRET-based biosensors by automating data analysis, detecting patterns in large datasets, and making predictions about biological processes. AI and ML can also be used to optimize FRET-based biosensors, making them more selective and specific and reducing false-positive results. With the increasing availability of large datasets and the growing need for high-throughput biosensing, the integration of AI and ML in FRET-based biosensors is expected to become increasingly important in the near future. The advancements in nanoscience and nanotechnology have greatly impacted the development and optimization of FRET-based biosensors. By using nanoscale materials, FRET-based biosensors can be made smaller, more sensitive, and more specific. The integration of nanoscale materials into FRET biosensors can also improve the stability and lifespan of these devices, making them more practical for long-term use. The use of nanoscale materials can increase the diversity of FRET-based biosensors, enabling the detection of a wider range of biological targets. Using fluorescent nanomaterials such as QDs as probes, it has become possible to monitor interaction in vitro as well as in vivo at ultra-low concentration even at the genetic level. With the continued progress in nanoscience and nanotechnology, it is expected that FRET-based biosensors will become even more advanced and play an increasingly important role in the study of biological processes and the development of new medical diagnostics. Despite having so many limitations and challenges, the recent era will be based on FRET-based biosensors/nano-biosensors touching every aspect of life all across the globe.

## Figures and Tables

**Table 1:** FRET-based immunosensors showing donor, acceptor, LOD, CR, and year-wise advancement in experiment.

Analyte	Causing/Biosensor	Donor	Acceptor	LOD	CR	Year	Ref.
Detection of PRRSV	Immunosensor	CdSe/ZnS	AuNRs	0.55 TCID_50_/mL	10^1^–3.5 × 10^4^ TCID_50_/mL	2022	[113]
Tumor biomarker	Immunosensor	First antibody labeled Si/NS-CDs	Second antibody labeled Au/AgNPs	NA	NA	2022	[114]
Detection of Staphylococcal enterotoxin B (SEB)	Immunosensor/Food Poisoning	QDs/SEB	SEB/Antibody	<1 ng/mL	20 ng/kg of body weight	2022	[115]
Progesterone hormone screening	Immunosensor	CDs	GO	13.8 nM	10–900 nM	2022	[116]
Detection of MAGE- A11 antigen	Immunosensor	GQD/anti-MAGE-A11	Graphene nanosheets	5.6 pg/mL	0.05–5 µg/mL	2022	[117]
OTA	Immunosensor	Amino methyl fluorescein AMF	AuNps	0.02 ng/mL	NA	2022	[112]
Dicofol	Immunosensor	Au/Ag NCs in PADs and Au/Ag NCs antigen donor in liquid	Au/NFs antibody	0.170 ng/mL in PADs and 0.185 ng/mL in liquid	NA	2022	[110]
Detection of helicobacter pylori	Immunosensor	FCDs	Graphene oxide	10 cells/mL	5–10^7^ cells/mL	2022	[111]
Detection of Lipovitelline of paralichthys Olivaceous	Immunosensor	afGQDs/anti-lv/mAb	rGO	0.9 pg/mL	0.001–1500 ng/mL	2022	[118]
Ochratoxin A (OTA) in agro products	Immunosensor/contamination of food	OTA	Nb (nanobody)	5 pg/mL in 5 min	NA	2020	[119]
Zn	Impaired immune system	CFP	YFP	5 µM	5–2 µM	2015	[98]
Aflatoxin B1	Immunosensor	Red QDs	Green QDs	0.13 pM	0.190.16 pM	2014	[120]

**Table 2:** FRET-based enzymatic sensors showing donor, acceptor, LOD, CR, and year-wise advancement in experiment.

Detection of	Causing/Biosensor	Donor	Acceptor	LOD	CR	Year	Ref.
Sensitive inkjet printing FRET biosensor	Enzymatic	5-FAM	QXL520	NA	NA	2018	[122]
Synthesized pyrimidine derivatives	Enzymatic	BSA/HSA	ANHP	NA	NA	2018	[130]
Monitoring of Enzyme activity at living cell surface	Enzymatic	Supramolecular Terbium (Tb)	Zwitterionic ligand coated QDs	40 pM	NA	2018	[131]
Neutrophil elastase	Enzymatic	CFP	YFP	NA	NA	2017	[125]
Thioredoxin reductase activity monitoring in cancer cells using biotin-CDs-naphthalimide biosensor	Enzymatic	Carbon dots	Naphthalimide	7.2 × 10−8 M	0–1 µg/mL	2017	[126]
Detection of paraoxon among organophosphate pesticides	Enzymatic biosensor	Tryptophan	IAEDANS fluorophores	NA	NA	2017	[132]
Cancer drug screening, therapeutic effect, and MMP-2 and caspase-3 evaluation	Enzymatic	FAM	Dabcyl	NA	NA	2015	[91]
Imaging of intracellular telomerase (in situ “on-off”) activity through telomerase-responsive MSN	Enzymatic	Mesoporous silica nanoparticle (MSN)	Black hole fluorescence quencher (BHQ	NA	NA	2013	[133]
MMP-2 and MMP-7 imaging through double-labeled FRET-biosensor	Enzymatic	AuNPs	Dye	NA	NA	2012	[15]
MMP-9, MMP-2 (proteases collagenase)	Enzymatic	QDs	Rhodamine	NA	5 µg/mL	2006	[57]

**Table 3:** FRET-based aptamer sensor showing donor, acceptor, LOD, CR, and year-wise advancement in experiment.

RNA junction	RNA aptamer	fluorescent aptamers Broccoli	Mango III	NA	NA	2021	[140]
DNA hybridization detection	DNA aptamer	Upconversion nanoparticles	SYBER green I	3.2 nM	7.6 nM	2018	[137]
Tracking RNA molecules in E. coli	Aptamer	Spinach/DFHBI-1T	Mango/YO_3_	NA	NA	2018	[141]
Ochratoxin A (OTA)	DNA aptamer	colloidal cerium oxide nanoparticles	graphene quantum dots	2.5 pg/mL	0.01–20 ng/mL	2017	[142]
Ochratoxin A (OTA)	DNA aptamer	colloidal cerium oxide nanoparticles	graphene quantum dots	2.5 pg/mL	0.01–20 ng/mL	2017	[142]

**Table 4:** Tissue-based FRET biosensor and various other biosensors showing donor, acceptor, LOD, CR, and year-wise advancement in experiment.

Acid active tumor targeting nanoplatform	Tissue Based	Camptothesin (CPT)	Maleimide thieoether	NA	500 µg/mL	2018	[86]
Fluorescent nano emulsion droplets (NEDs)	Tissue Based	1% of Cy5.5LP	1% of Cy7.5LP	NA	NA	2016	[143]
Detection of doxorubicin	Other FRET sensor	Carbon dots	Doxorubicin	13.8 nM	0.8 mg/mL	2017	[150]
Thermos sensitive nanoscale monitoring	Thermal Biosensor	FITC	Rhodamine B	10 nM	NA	2017	[155]
Multistep FRET system	Other FRET sensor	Py or pyrene	Acf or acriflaviv	NA	Na	2016	[157]
